# Structural Differences between the *Streptococcus agalactiae* Housekeeping and Pilus-Specific Sortases: SrtA and SrtC1

**DOI:** 10.1371/journal.pone.0022995

**Published:** 2011-08-30

**Authors:** B. Khare, V. Krishnan, K. R. Rajashankar, H. I-Hsiu, M. Xin, H. Ton-That, S. V. Narayana

**Affiliations:** 1 Center for Biophysical Sciences and Engineering, University of Alabama at Birmingham, Birmingham, Alabama, United States of America; 2 University of Texas Health Science Center, Houston, Texas, United States of America; 3 NE-CAT, Advanced Photon Source, Argonne National Laboratory, Chicago, Illinois, United States of America; University of Birmingham, United Kingdom

## Abstract

The assembly of pili on the cell wall of Gram-positive bacteria requires transpeptidase enzymes called sortases. In *Streptococcus agalactiae*, the PI-1 pilus island of strain 2603V/R encodes two pilus-specific sortases (SrtC1 and SrtC2) and three pilins (GBS80, GBS52 and GBS104). Although either pilus-specific sortase is sufficient for the polymerization of the major pilin, GBS80, incorporation of the minor pilins GBS52 and GBS104 into the pilus structure requires SrtC1 and SrtC2, respectively. The *S. agalactiae* housekeeping sortase, SrtA, whose gene is present at a different location and does not catalyze pilus polymerization, was shown to be involved in cell wall anchoring of pilus polymers. To understand the structural basis of sortases involved in such diverse functions, we determined the crystal structures of *S. agalactiae* SrtC1 and SrtA. Both enzymes are made of an eight-stranded beta-barrel core with variations in their active site architecture. SrtA exhibits a catalytic triad arrangement similar to that in *Streptococcus pyogenes* SrtA but different from that in *Staphylococcus aureus* SrtA. In contrast, the SrtC1 enzyme contains an N-terminal helical domain and a ‘lid’ in its putative active site, which is similar to that seen in *Streptococcus pneumoniae* pilus-specific sortases, although with subtle differences in positioning and composition. To understand the effect of such differences on substrate recognition, we have also determined the crystal structure of a SrtC1 mutant, in which the conserved DP(W/F/Y) motif was replaced with the sorting signal motif of GBS80, IPNTG. By comparing the structures of WT wild type SrtA and SrtC1 and the ‘lid’ mutant of SrtC1, we propose that structural elements within the active site and the lid may be important for defining the role of specific sortase in pili biogenesis.

## Introduction

In Gram-positive bacteria, cell wall-anchored surface proteins are virulence factors and are essential for host-cell adhesion, nutrient acquisition and many other functions that facilitate pathogen survival in a hostile environment [1], [2]. Anchoring of these surface proteins to the cell wall peptidoglycan requires transpeptidase enzymes called sortases, a product of an *srt* (surface protein sorting) gene [3], [4]. The substrates of sortases are protein precursors with an N-terminal signal peptide and a C-terminal cell wall sorting signal (CWSS), consisting of the LPXTG motif, followed by a segment of hydrophobic and basic residues [1]. The membrane-associated sortase recognizes the LPXTG motif of the substrate protein, cleaves between the Thr and Gly, and forms an acyl-enzyme intermediate [5]. Nucleophilic attack by the amino group of a peptidoglycan precursor, lipid II, resolves the acyl intermediate and releases the enzyme [6]. The subsequent transglycosylation and transpeptidation reactions incorporate the surface protein into the peptidoglycan and decorate the bacterial cell surface with covalently bound proteins [6].

Like surface proteins, pilins of Gram-positive bacteria harbor an N-terminal signal peptide and a C-terminal CWSS. Pilins are covalently assembled [7], [8] into thin and flexible fibers called pili, which cover the bacterial cell surface and participate in many functions, including host-cell attachment and biofilm formation [9]. The mechanism of Gram-negative pili biogenesis has been extensively investigated [10], [11] and involves chaperones that are non-covalently associated with the cell wall [12], [13]. In contrast, the Gram-positive pili, present in microorganisms such as *Actinomyces naeslundii* [14], [15], *Corynebacterium diphtheriae* [7], [16], *Bacillus anthracis* [17], *Streptococcus agalactiae* [18], [19], [20], *Streptococcus pyogenes* [21], and *Streptococcus pneumoniae* [22], [23], are covalently attached to peptidoglycans and require multiple sortases of different types for assembly and anchoring [8], [24], [25], [26]. The Spa-type pilus of *C. diphtheriae* is the best studied; it is encoded by the *spaA-spaB-srtA-spaC* locus that is composed of three pilins: SpaA forming the shaft, SpaC at the tip, and SpaB along the pilus structure and at the base [7]. In addition to the CWSS, SpaA contains a pilin motif that is involved in the sortase-catalyzed covalent cross-linking between SpaA subunits. The pilin-specific sortase, SrtA, is the only enzyme required for the polymerization of SpaA pilins, which is terminated when SpaB enters at the pilus base; the housekeeping sortase, SrtF, whose gene is located in a different chromosomal region, then catalyzes the cell wall anchoring of the assembled pilus. A study by Swaminathan et al. [27] has shown that the function of *C. diphtheriae* SrtF may be limited to the cell wall anchoring of pilins, whereas the *C. diphtheriae* SrtA sortase has dual functions, that is, polymerizing and cell wall anchoring activities, although its cell wall anchoring activity is less efficient than that of SrtF. Intriguingly, by swapping the LPXTG motif of SpaB with that of SpaA, SrtA is able to anchor its pilus polymers to the cell wall in the absence of SrtF, indicating that the LPXTG motif may determine the substrate specificity.

Similar to corynebacterial pili, the *S. agalactiae* (Group B streptococcus, GBS) pili are heterotrimeric, and the individual components are promising candidates for pilus-based vaccines [28], [29]. Historically known for life-threatening neonatal infections, such as sepsis, pneumonia, and/or meningitis [30], GBS also causes invasive disease in pregnant and non-pregnant adults [31], [32], [33]. GBS disease in the elderly manifests as bacteremia and as peritoneal skin and soft tissue diseases [33]. Two pilus islands (PI-1 and PI-2a) have been identified in strain 2603V/R (serotype V), which is the strain responsible for most GBS infections in the elderly. The PI-1 cluster encodes three pilins, the major pilin GBS80 (or SAG0645) and two minor pilins GBS52 (SAG0646) and GBS104 (SAG0649), and the two sortases SrtC1 and SrtC2 (or SAG0647 and SAG0648, respectively) [18], [20]. The PI-2a locus encodes three pilins, the major pilin GBS59 and two minor pilins GBS67 and GBS150, in addition to the two sortases SrtC3 and SrtC4. The *srtA* locus encodes the housekeeping sortase SrtA, and it resides elsewhere in the genome. Interestingly, though either SrtC1 or SrtC2 is sufficient for the polymerization of GBS80, the incorporation of the GBS52 and GBS104 minor pilins into the pilus structure requires SrtC1 and SrtC2, respectively. Consistent with the two-step mechanism of pilus assembly described above for corynebacterial pili (i.e., pilus polymerization preceding cell wall anchoring), the housekeeping SrtA sortase has been shown to be involved in cell wall anchoring of the PI-2a pilus polymers via the minor pilin, GBS150 [34].

The pilus-specific sortases and the housekeeping sortase belong to class C and A sortases, respectively, and their nomenclature is based on their function and substrates [35]. Sortases of the same class from different pathogens exhibit limited primary sequence identity (∼21%), which is even lower among different types from the same pathogen [36], [37], [38]. The available crystal structures of class A and C sortases from different pathogens [39], [40], [41], [42], [43] reveal similar overall structures, with differences in the active sites and surrounding loop regions that often dictate the specificity for proteases. The first crystal structures of sortases, *S. aureus* sortase A (SASrtA) and sortase B (SASrtB) [39], [40], displayed novel catalytic residue arrangements with an unconventional distance (>3.8 Å) between the nucleophilic Cys and the conserved (and presumed catalytic) His. Based on the substrate and inhibitor complex structures of SASrtA and SASrtB, respectively [39], [40], Zong *et al.* have proposed that a conserved and proximally positioned Arg will partner with the catalytic Cys for both the proteolysis of the LPXTG motif and the transpeptidation reaction between the acylated sortase and the amino group of lipid II. Site-directed mutagenesis and enzymatic assays have shown that Arg and His are absolutely required for catalysis [44], [45], and Arg might help in the substrate binding and stabilization of the acyl-enzyme intermediate, analogous to an ‘oxyanion hole’ in serine proteases [46]. In this report, we present the crystal structure of GBSSrtA and show that the catalytic site is more open and extended in comparison to SASrtA. In addition, we reveal the spatial arrangement of putative catalytic residues, similar to that seen in *S. pyogenes* sortase A (SPYSrtA) though different from SASrtA.

The crystal structures of three pilus-specific sortases from *Streptococcus pneumoniae* (SPNSrtC1–C3) [43], [47] have revealed an N-terminal loop, termed the ‘lid’, positioned close to the catalytic residues in the respective putative substrate binding sites; it has been proposed that the opening/closing of the lid facilitates the accessibility to the binding pocket. Manzano *et al.* (2009) later showed that mutation of the anchor residues in the lid did not affect substrate recognition or acyl-intermediate formation; instead, it adversely affected the stability and efficiency of the enzyme [48]. The mechanism by which the lid promotes either substrate accessibility or enzyme stability is not clear yet. Furthermore, structure-function relationships of class C and class A sortases from a single strain of the same pathogen have not been studied so far.

In our previous report [49], we described the recombinant expression, purification, crystallization and diffraction data collection statistics for the pilus-specific GBS sortase C1 (GBSSrtC1; type I, II and III, corresponding to three different space groups) and the housekeeping GBS sortase A (GBSSrtA) from the SAG 2603 V/R strain. Here, we present two crystal structures of the GBSSrtC1 (type II and III) determined by molecular replacement methods and that of GBSSrtA determined by SAD-MR phasing. In addition, we also present the crystal structure of a lid mutant of GBSSrtC1. Toward an understanding of the factors that confer lid-associated enzyme stability we identified structural features within the putative active site of GBSSrtC1 that may explain the presence of the lid and its proposed role of maintaining enzyme integrity. Lastly, we detail some of the structural differences between GBSSrtA and GBSSrtC1 and discuss the structure-function relationships of the two classes of GBS transpeptidase enzymes.

## Results

### Crystal Structure of GBSSrtC1

**Figure 1:**
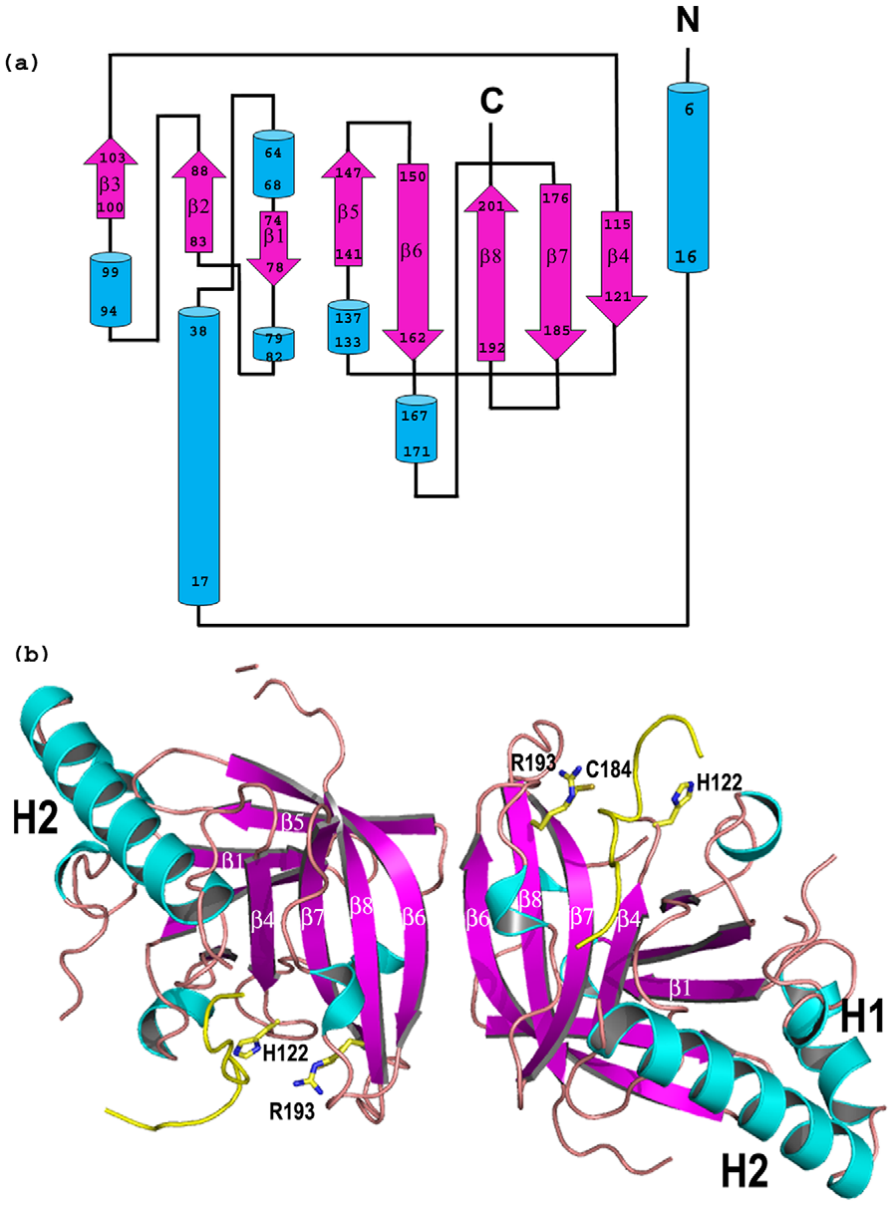
The topology and secondary structural elements of GBS Sortase C1. (a) The helices are represented as blue cylinders, β-strands as arrows in pink color and loops as lines in black color (b) Individual GBSSrtC1 is made of an eight-stranded beta-barrel fold and the interface of the dimer present in the asymmetric unit, is formed by the β6 strands. The N- and the C- termini are marked. Helices H1 and H2 flanking the barrel core lie on the opposite sides of the dimer interface. The catalytic residues (C184, R193 and H122) are shown in sticks and the lid region is shown in yellow.

**Figure 2:**
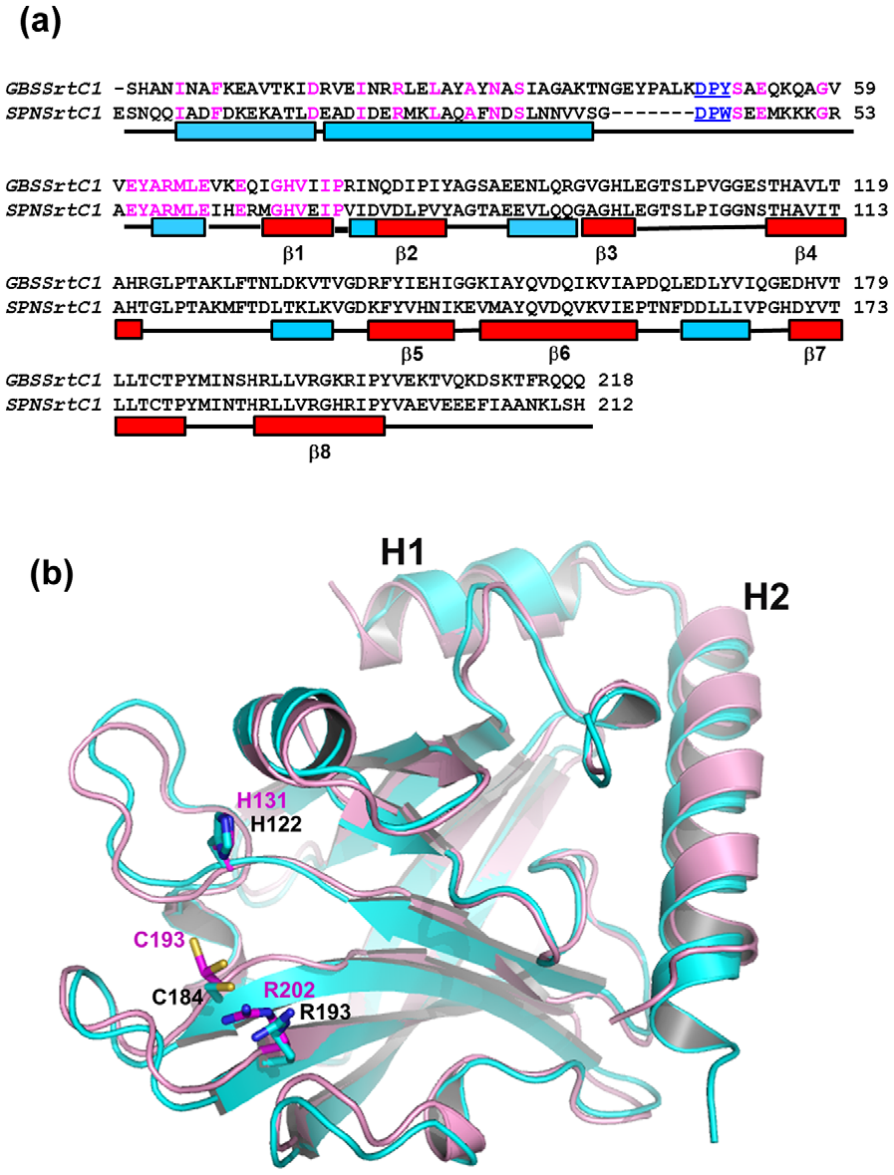
Similarities between of GBS SrtC1 and SPN SrtC1 (PDB code- 2W1J). (a) In the primary sequence alignment achieved by secondary structural element superposition, the conserved DP(Y/W) motif is highlighted in red, and identical residues in pink color. The secondary structural elements in GBSSrtC1are presented as red boxes for β-strands and blue for helices and loops as black lines and depicted below the sequence. (**b**) The backbone superposition of GBSSrtC1 (cyan) and SPNSrtC1 (magenta) is shown and the corresponding catalytic residues are depicted as sticks and labeled accordingly.

**Figure 3:**
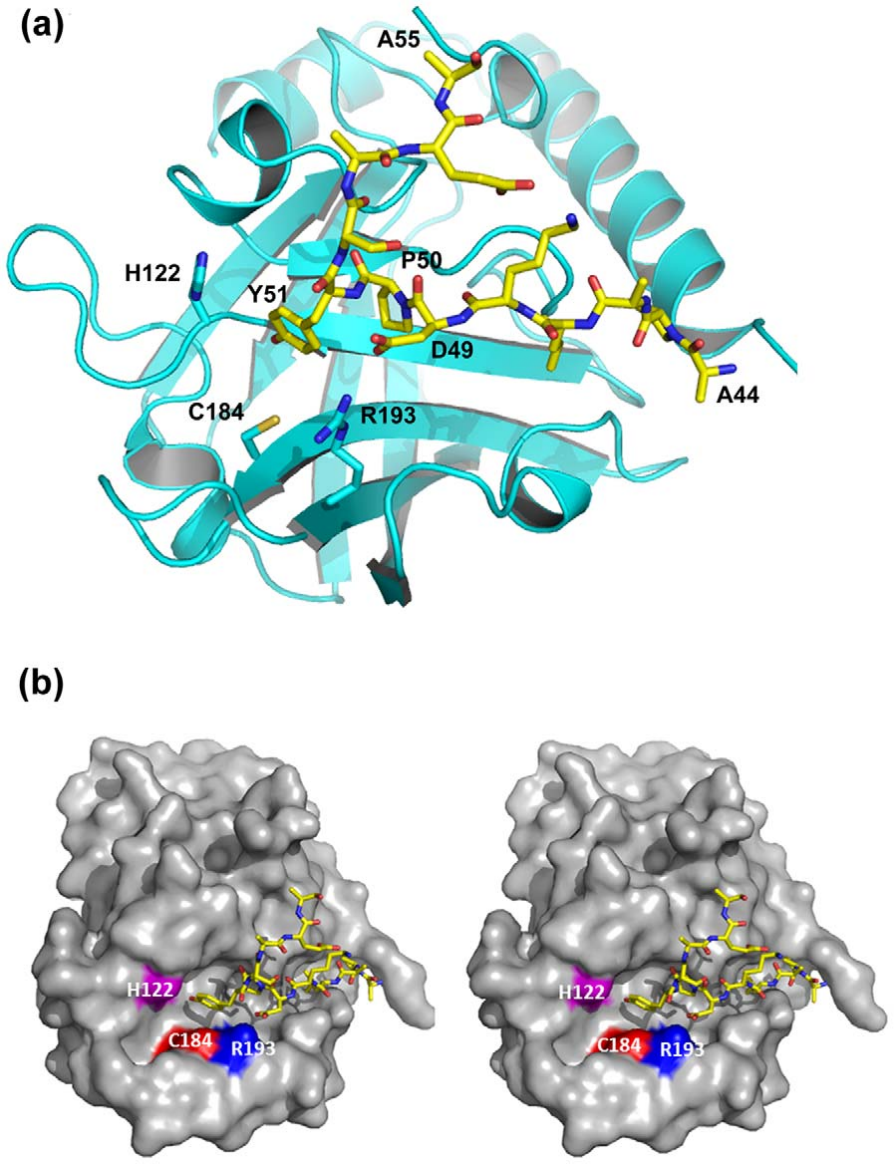
The putative active site of GBSSrtC1 with the protecting ‘lid’ (a). Residues 44–55 are seen positioned in the putative binding pocket close to the catalytic residues in one of the molecules of the GBSSrtC1 Type II dimer. Tyr51, Asp49 and Leu47 residues anchor the lid in position. (**b**) A Stereo view of GBSSrtC1 monomer in surface representation shows an elongated putative active site groove and the ‘lid’ (yellow, sticks) seen occupying the length of the groove. Catalytic residues Cys184 (red), His122 (magenta) and Arg193 (blue) are marked.

The two molecules of the dimer, related by a non-crystallographic 2-fold axis, in the asymmetric unit of both the type II and III crystals of the GBSSrtC1 display the typical sortase fold made of an eight-stranded beta-barrel (Figure 1a). The barrel core is flanked by two long N-terminal helices (H1 and H2, Figure 1b), positioned approximately at 75° to each other and away from the dimer interface, which is formed by β6 strands and β4/β5 loops. The major differences between the four molecules are localized at the peripheral regions, such as the H2/β1, β2/β3, β4/β5 and β6/β7 loops and the C-terminus. Subtle differences, such as one single-turn α-helix in the β1/β2 loop present only in chain B of both dimers, and a four-turn and two-turn H1 helix in the type III and II structures, respectively, may explain the plasticity in the sortase structural features, the presence of multimers in the asymmetric unit and the observed multiple crystal-packing arrangements. Based on the SASrtA-substrate complex structure [40], we suggest an elongated cleft that widens towards the catalytic Cys184 residue on one end is the putative active site of GBSSrtC1, with the β7, β8 and β4 strands as its floor and the β6/β7, β3/β4 and the β2/helix5 loops as the walls of this binding pocket (Figure 2b and 3b).

Both GBSSrtC1 and GBSSrtC2 of SAG2603 V/R were shown to be capable of polymerizing GBS80, similar to SPNSrtC1 and SPNSrtC2 of *Streptococcus pneumoniae* strain TIGR4, which show cross-specificity for the major pilin RrgB [36], [50]. The primary sequence (Figure 2a) and the crystal structures of GBSSrtC1 and SPNSrtC1 (Figure 2b) share similarities (rmsd = 1.8 Å over 177 CA atoms), although variations were found in the conformations, orientations and composition of the loops connecting the β strands in the active site, the N-terminal helical domain and both termini.

Residues 44–55 and 44–54 in type II (A and B chains, respectively) and 44–51 and 46–53 in type III (chain A and B, respectively), which cover the putative active site from one-end to the other, form the lid region in GBSSrtC1. Despite the high flexibility and temperature factors (80–100 Å^2^), residues 46–51 were consistently positioned in all four of the active sites (Figure 3a and 3b). Unlike the SPNSrtC1–C3 crystal structures, the hinge region between the H2 helix and the lid and the link between the lid and the β1 strand were not observed in the GBSSrtC1 structures, possibly due to their high flexibility.

**Figure 4:**
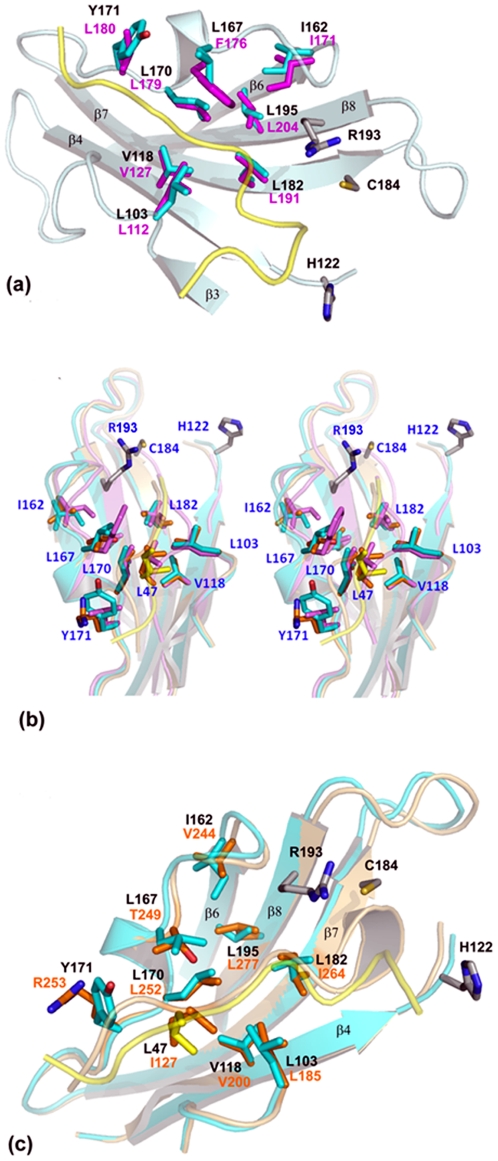
The conserved hydrophobic pockets and the covering ‘lid’ regions. (a) The GBSSrtC1 (Type II chain A) active site (pale blue) has the lid (yellow) positioned over the hydrophobic pocket of the putative active site. The residues comprising this pocket are shown in sticks (GBSSrtC1 residues in cyan, labeled in black and SPNSrtC1 residues and labels in magenta; the numbering of the residues is according to the respective structures). A single conformation of SPN Leu191 is shown. The catalytic residues of GBSSrtC1 (sticks) are also shown. (**b**) Stereo view of the putative active sites of GBSSrtC1 (cyan; lid in yellow), SPNSrtC1 (violet) and AORSrtC1 (orange) looking down toward the hydrophobic pocket. The HB-lid in all three pilus-specific sortases inserts into the hydrophobic pocket and is spatially conserved. (**c**) The hydrophobic pocket residues of GBSSrtC1 (cyan) and AORSrtC1 (orange) are highly conserved. The two positions corresponding to Tyr171 and Leu167 of GBSSrtC1 are different in AORSrtC1 but spatially conserved. Also shown are the HB-lid residues of the two enzymes.

Analogous to the Asp58, Pro59 and Trp60 residues of the conserved DP(W/F/Y) motif in SPNSrtC1 [43], [48], Asp49, Pro50 and Tyr51 of GBSSrtC1 anchored the lid in its active site. In addition, the preceding leucine residue (Leu47) was observed inserted into a highly hydrophobic pocket in the active site present below the lid. The residues in the hydrophobic pocket included Val118 (on the β4 strand), Ile162 (at the edge of the β6 strand) and Tyr171 (on the β6/β7 loop) (Table 1; Figure 4a). Similar to the Leu47 of GBSSrtC1, Val55, Ile84 and Ile72 are the hydrophobic residues from the ‘lids’ (hereafter referred to as HB-lid) inserted into the hydrophobic pockets in SPNSrtC1, SPNSrtC2 and SPNSrtC3, respectively. Though the conformations of the lid regions varied somewhat, the HB-lid residues occupied spatially similar positions in all of the five class C pilus-specific sortase structures (Figure 4b), along with the corresponding pocket residues below the lid, conserved both in identity and spatial position (Table 1; Figure 4b). Only the two residues Leu167 and Tyr171 in GBSSrtC1 presented by the β6/β7 loop are variable in identity, hydrophobic character or spatial conservation among pilus-specific sortases. A similar trend in the conservation of the hydrophobic pocket residues and the HB-lid residue was also observed for the *Actinomyces oris* sortase C1 (AORSrtC1) [51] (Table 1, Figure 4c). Interestingly, the conformation of the AORSrtC1 lid is most similar to the SPNSrtC2 lid; the hinge between the lid anchor and the β1 strand that has been observed to form a helix in AORSrtC1 was disordered in SPNSrtC2. The HB-lid residue in AORSrtC1 is Ile127, spatially analogous to the SPNSrtC2 Ile84.

**Table 1:** The residues forming the hydrophobic pocket and the hydrophobic lid anchor residue in the pilus-specific sortases.

GBSSrtC1	SPNSrtC1(2W1J)	SPNSrtC2(3G66)	SPNSrtC3(2W1K)	AORSrtC1(2XWG)
Leu103	Leu112	Leu140	*Val125	Leu185
Val118	Val127	Val155	Val140	Val200
Leu167	*Phe176	*(Phe204)	*(W189)	*Thr249
Leu170	Leu179	*Val207	Leu192	Leu252
Leu182	Leu191	Leu219	*Ile204	*Ile264
Leu195	Leu204	Leu232	Leu217	Leu277
Ile162	Ile171	*Val199	Ile184	*Val244
Tyr171	*Leu180	*Leu208	**	*Arg253
**Leu47**	§ **Val55**	§ **Ile84**	§ **Ile72**	§ **Ile127**

**The PDB codes for different structures are also given.**

The lid anchor residues are given in bold.

*Identity of this residue is different from the corresponding residue in GBSSrtC1.

**This position is occupied by Glu193 and its side chain is spatially conserved with Tyr171 side chain.

Residues in parentheses indicate that the residue does not occupy the same spatial position as the structurally corresponding residue in GBSSrtC1.

§ While the Cα's of HB-lid residues do not coincide with Leu47 upon superposition, the orientation and spatial position of the sidechains over the hydrophobic pocket is conserved.

**Figure 5:**
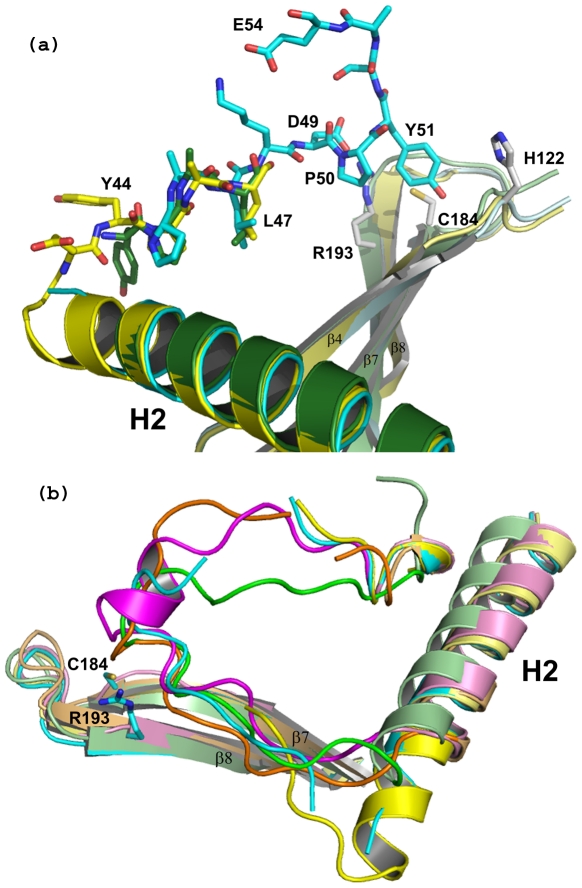
Comparison of GBSSrtC1 lid mutant with other pilus specific sortase structures. (a) The two molecules of GBSSrtC1-lidM (yellow and green) show differences in the hinge region but the minimal parts of the lid are ordered till Leu47, as in the GBSSrtC1 structure (cyan). The lid region of the wt GBSSrtC1 structure is shown in cyan. (**b**) Superposition of the lid and hinge regions of the GBSSrtC1 (cyan), GBSSrtC1-lidM (yellow), SPNSrtC1 (magenta), SPNSrtC2 (orange) and SPNSrtC3 (green) structures in relation to the corresponding catalytic residues in GBSSrtC1.

A few residues of the hinge regions at both ends of the lid in all of the GBSSrtC1 monomers were disordered, compared to the fully observed hinge regions in SPNSrtC1, SPNSrtC3 and, to some extent, in SPNSrtC2. Although the lids of the pilus-specific sortases exhibit high temperature factors, remarkably, the observed minimal parts of the ‘lid to β1 strand’ hinge in GBSSrtC1 followed a conformation that was essentially similar to that observed for SPNSrtC1–C3 (Figure 5b). Minor variations in lid conformations, as observed between the four GBSSrtC1 molecules, also have been found to occur between SPNSrtC1–C3 crystal structures.

The three catalytic residues Cys184, His122 and Arg193 of GBSSrtC1 and the corresponding residues of SPNSrtC1 (chain A) displayed similar spatial arrangements [43]; Cys184 and Arg193 were present on the β7 and β8 strands, respectively, whereas His122 was on a loop extending from the β4 strand. Cys184 in the GBSSrtC1 monomers displayed a single conformation with its sulfhydryl pointing toward Arg193, which adopted a similar conformation in all of the monomers due to salt-bridge interactions with Asp49 of the lid DP(W/F/Y) motif. Arg193 also formed a weak hydrogen bond with the backbone carbonyl of Asn190, except in the type II chain A structure.

### Crystal Structure of GBSSrtC1-lid Mutant (GBSSrtC1-lidM)

Crystal structures of a number of the pilus-specific C type sortases have been reported thus far [43], [47], [51]; however, no structures are available for their substrate complexes. Our attempts to co-crystallize pilin sorting-motif peptides (of different lengths) with GBSSrtC1 were unsuccessful. Because the DP(W/F/Y) motif anchors the lid in all of the putative active sites of GBSSrtC1 monomers, we reasoned that the substitution of the ‘KDPYS’ region with IPNTG, the sorting motif of the shaft pilin, GBS80, may present us a view of the substrate-bound active site. Our previous experience with an active SASrtA suggested that, in the absence of the second substrate, the sorting peptide undergoes slow hydrolysis when soaked into crystals. Therefore, we generated a Cys184Ala mutant of GBSSrtC1, containing the lid mutation (KDPYS to IPNTG), and used this mutant for crystallization.

The association of the two GBSSrtC1-lidM monomers observed in the triclinic unit cell, related by a non-crystallographic 2-fold axis, is identical to that observed for the type II and III GBSSrtC1 dimer association. The two monomers of GBSSrtC1-lidM, with an rmsd of 0.63 Å over 164 atoms, display differences around the active site region. Molecule A showed a continuous electron density for the hinge region between H2 and the lid, and this formed an extra helical turn. In chain B, residues 39–43 of this hinge remained disordered. The position of the next ordered residue, Tyr44, differed considerably between the monomers and may be due to the flexibility of this region.

Both GBSSrtC1-lidM monomers lacked electron density for the introduced ‘IPNTG’ motif. The lid in both chains was visible up to Leu47, which points inwards into the hydrophobic pocket, positioned precisely as in GBSSrtC1 structures (Figure 5a). The average overall temperature factors of monomers A and B were 40.55 and 48.48 Å^2^, respectively. The B factors of the consecutive residues in the lid region increased sequentially, with higher values for the respective B chain residues, but with considerably lower values for Leu47 of both monomers. The low B factors for Leu47 in GBSSrtC1-lidM and its surrounding hydrophobic environment may suggest that the spatial disposition of Leu47 acts as an anchor to the lid.

The disposition of Ala184 and the catalytic His122 and Arg193 remained unaffected in the absence of the DP(W/F/Y) motif; however, the orientation of Arg193 side chain was slightly different from that seen in the GBSSrtC1 structures.

### Crystal Structure of GBSSrtA

The crystallized GBSSrtA is a truncated recombinant, lacking 81 and 9 residues at the N- and C-termini, respectively. The crystals belonging to the C2 space group diffracted weakly to 2.9 Å. Useful data were processed and scaled up to 3.1 Å (R_merge_ = 11%). Despite the poor data quality, the initial electron density map calculated using MR-SAD phases to 3.2 Å was of good quality, suitable for chain tracing and had distinct density for the residue side chains. The quality of the map was further improved by bulk-solvent correction in addition to anisotropic scaling [52], non-crystallographic symmetry averaging and, subsequently, by iterative cycles of restrained refinement and model building.

**Figure 6:**
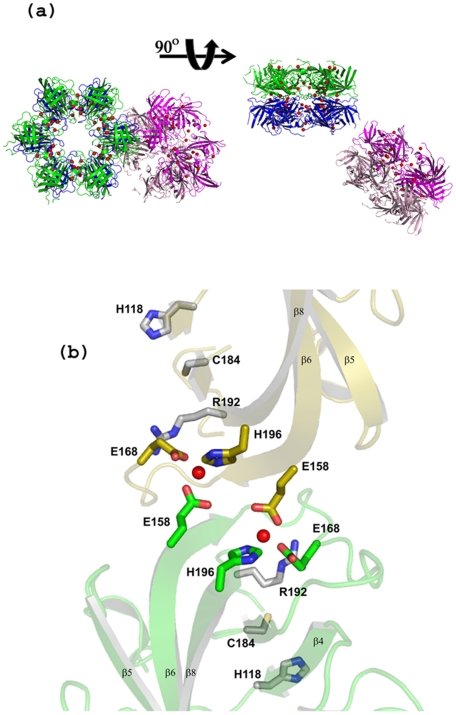
GBS housekeeping sortase SrtA crystal packing. (**a**) Two views of the 18 GBSSrtA molecules in the asymmetric unit. The three hexameric rings X (green), Y (blue) and Z (pink) are shown, the X and Y hexamers formi a donut-like oligomer and the third Z hexamer forms another such oligomer with a symmetry-mate (pale pink). The zinc atoms are shown as red spheres. (**b**) The inter-hexameric dimer of GBSSrtA: showing two zinc coordination sites similar to those observed in zinc binding proteins. Each zinc ion is coordinated by two Glutamic residues donated by the two neighbors and one Histidine residue. The catalytic residues in relation to the zinc coordination sites are identified.

The 18 GBSSrtA molecules in the asymmetric subunit are packed into three hexamers, X (X1, X2, ‥X6), Y (Y1, Y2,…Y6) and Z (Z1, Z2,‥Z6) (Figure 6a). X and Y are related to one another by a non-crystallographic 2-fold axis, resulting in a donut-like multimer; and Z formed another such multimer with a symmetry-related hexamer. The molecules in X and Y are arranged in clockwise and counter-clockwise directions, respectively, with X1 juxtaposed against Y6 related by a non-crystallographic 2-fold axis. The residue numbering scheme we followed is as follows: residue numbers for six individual monomers in X hexamer are incremented by 1000; thus C1184 of X1, C2184 of X2, C3184 of X3,……C6184 of X6 are equivalent. Similar 1000 increments are done for residue numbers of the six monomers in Y and Z hexamers, respectively.

All 18 molecules display a similar sortase fold; however, the β7 and β6 strands in some monomers are much longer (e.g. in X3, X6, Y2, Y4 etc.) than in others such as Z2 and Z5. Three short helices, one each between β1/β2, β2/β3 and β4/β5 strands, observed in most of the monomers, are absent in few monomers such as X3 and Y1.

The 18 GBSSrtA monomers in the asymmetric unit are held together by an elaborate network of zinc ions (Figure 6a): 36 of them are localized at dimeric interfaces. The inter-hexameric dimer interface (e.g., X1 and Y6) is formed by the β6 strand, the β6/β7 loop, the termini and the zinc sites coordinated to Glu158 from one molecule and Glu168 and His196 from the other. Hence, the two juxtaposed monomers (e.g., X1 and Y6) are held together by non-crystallographic 2-fold interactions. Six such pairs of molecules were held in place by Zn ions between adjacent monomers (i.e., X1 and X2 or X6 and X1) of the same hexamer (Figure 6b). The second, intra-hexameric coordination site (i.e., X1 and X2) consisted of the catalytic His118 of X1 and His136 of X2; His136 of X1 formed similar coordination with His118 of X6. These sites resemble the protein-interface coordination sites found in many zinc-binding proteins [53], [54]. Extensive crystallization trials in the presence of EDTA, conducted on a number of GBSSrtA constructs, failed to yield diffraction-quality crystals, which we obtained only with the present recombinant enzyme using zinc salts (i.e., Zn [SO_4_]_2_ or Zn [O_2_CCH_3_]_2_) in the reservoir solution.

**Figure 7:**
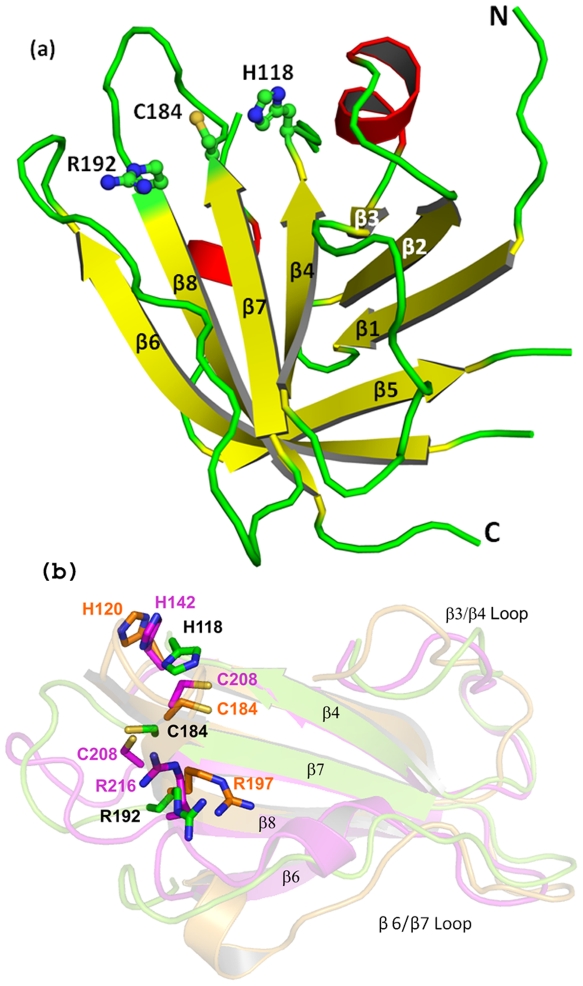
Comparison of GBSSrtA and other housekeeping sortases. (**a**) Cartoon representation of GBSSrtA secondary structure; the β-strands are depicted in yellow color, α-helices are in red and loops are in green color. The side chains of catalytic residues Cys184, His118, and Arg192 are represented as sticks. (**b**) Superposition of the GBSSrtA (green), SPYSrtA (magenta) and SASrtA (orange) catalytic residues and the surrounding contributing regions.

The loops protruding into the central cavity of the donut-shaped multimer are typically different between the monomers. The putative active site groove of SASrtA is equally wide on either end, whereas the long, inwardly bent β7/β8 loop blocked the groove at the catalytic end. In sharp contrast, the β7/β8 loop of GBSSrtA, fully defined except in molecules X2–5, Y3–4, Y6, Z1 and Z6, is much shorter and in an “open” conformation. With an equally wide active site, the catalytic Cys184 and Arg192 of GBSSrtA reside at the edges of the anti-parallel β7 and β8 strands, respectively (Figure 7a). The Cys184 sulfhydryl in all of the monomers is pointing away from His118, hence, the distance between them is greater than 3.8 Å. However, unlike for SASrtA, His118 is present on a loop at the C-terminal end of the β4 strand and as a ligand for zinc coordination it showed a near-identical disposition in all of the monomers, except in Y4, where it moved closer toward the β7 strand that hosted Cys184. In X1, the Cys184 sulfhydryl oriented directly at Phe131 of X2, which is stacked between its Pro187 and His118 aromatic rings. The backbone carbonyl of His166 on the β6/β7 loop is hydrogen bonded with the catalytic Arg192 side chain and stabilized the latter. Interestingly, the imidazole ring of the H166 is stabilized by its interactions with a Zn atom positioned between the hexamers. The GBSSrtA Arg192 is spatially conserved in all of the monomers, and has comparable disposition to Arg197 of SASrtA.

The overall architecture of GBSSrtA is closer to SPYSrtA, the only other pilus-anchoring sortase (A type) crystal structure available. A key difference is at the C-terminus beyond the β8 strand, which is 20 residues long, with two small helical segments, and reached the backside of SPYSrtA active site. The absence of such long C-terminus (as the derivative was only 9-residues long and truncated for expression) resulted in a flexible β4/β5 loop for GBSSrtA. Despite being part of the dimer interface, the position of the GBSSrtA β6/β7 loop, which is proposed to dictate substrate specificity for sortases [55] is identical to that in SPYSrtA. In both the structures, the catalytic His resided at the N-terminal end of the β4/β5 loop and the dispositions of side chains are almost similar but slightly displaced due to its coordination with Zn in GBSSrtA. Orientation of catalytic Cys side chain in GBSSrtA is similar to that in SPYSrtA: both oriented away from the catalytic His (Figure 7b). The catalytic Arg lay at the same place on the β8 strand in both GBSSrtA and SPYSrtA.

### Comparison of GBSSrtA and GBSSrtC1 active sites

**Figure 8:**
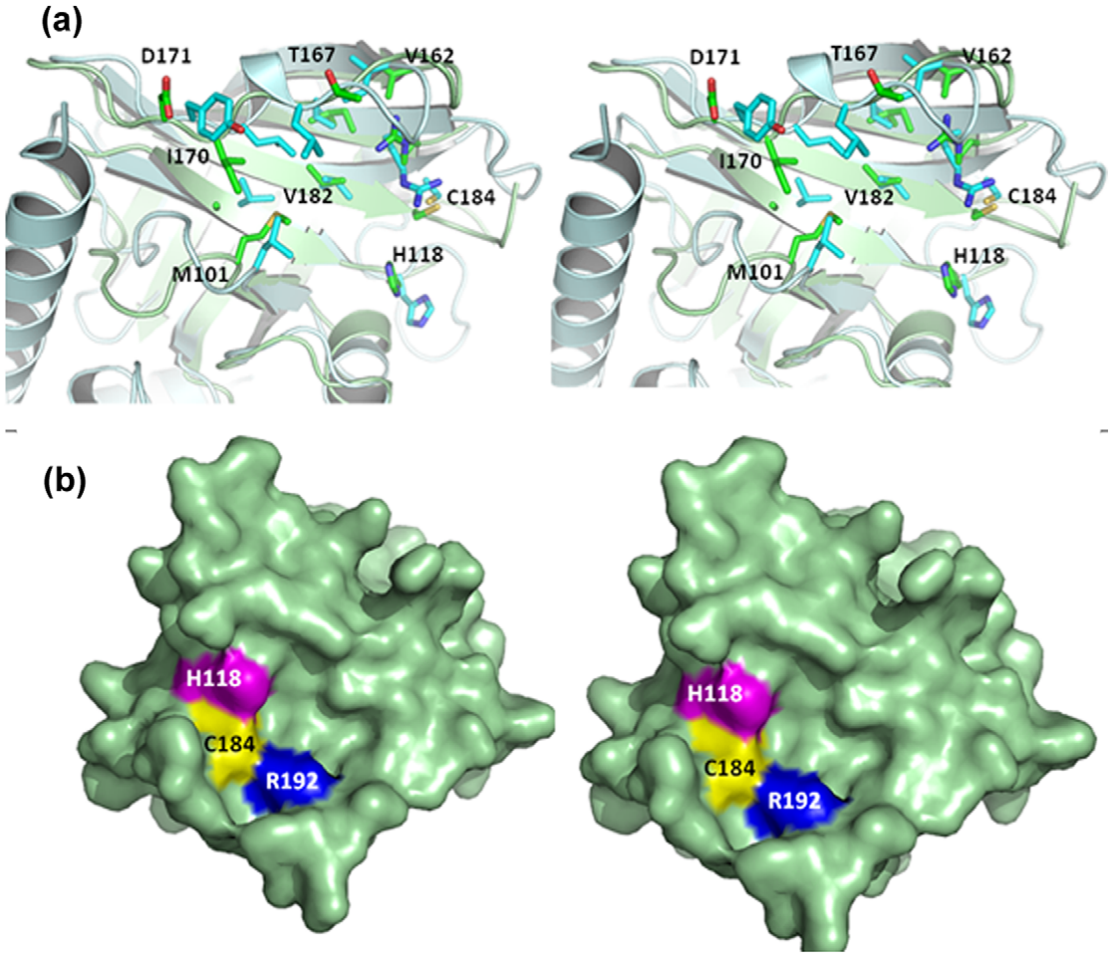
Comparison of GBSSrtA and GBSSrtC1 structures. (**a**) The superposition of the GBSSrtA (green) and GBSSrtC1 (cyan) active sites shows the respective catalytic and hydrophobic pocket residues. Corresponding residues in GBSSrtA shows a less conserved pattern of hydrophobic residues between the housekeeping sortase and the pilus-specific sortase. (**b**) Stereo view of the surface representation of GBSSrtA shows a putative active site groove, different from GBSSrtC1. The catalytic Cys184, His118 and Arg192 are colored yellow, magenta and blue, respectively.

The beta-strands that formed the floor of the putative binding pockets in GBSSrtA and GBSSrtC1 varied in length resulting in slight differences in the disposition of the respective catalytic residues. Hexamers X, Y and Z have an overall temperature factor of 54.5, 62.6 and 52.1 Å^2^, respectively. Based on its lowest overall B value within the hexamer, ordered catalytic residues and positive density for all residues, we selected molecule X2 of GBSSrtA for comparison with the type II chain A molecule of GBSSrtC1. The distance of Cys from Arg and His (residues C184, R192 and H118, respectively in X2) is 4.9 Å each (the average over 18 molecules is 4.5 Å and 4.9 Å, respectively). The corresponding distances in the GBSSrtC1 molecule (residues 184, 193 and 122, respectively) are 4.5 Å (the average over 4 molecules is 4.5 Å) and 7.9 Å (the average for 4 molecules is 7.7 Å), respectively suggesting that Cys184 in GBSSrtC1 is closer to the respective catalytic Arg residue, rather than His, compared to GBSSrtA. The directions of the sulfhydryls are almost opposite to each other in the two enzymes (Figure 8a). The Arg193 side chain is held in place by Asp49 in the lid of GBSSrtC1, and Arg192 of GBSSrtA is held in place by the main chain carbonyl of His166 from the β6/β7 loop.

The β7/8/4 strands and the β6/β7 and β3/β4 loops contributed hydrophobic residues to the putative active sites. Except for Val118 and Thr120 of the β4 strand in GBSSrtC1, which are replaced with alanines in GBSSrtA, the hydrophobic (mostly identical) and polar residues (similar in polarity) are spatially conserved in both of the sortase active sites. Located on the β3/β4 loops, Met101 and Leu103 in GBSSrtA and GBSSrtC1, respectively, occupied the same place and equally covered the hydrophobic residues of the active site. Interestingly, the hydrophobic patch in GBSSrtC1, which formed the left side wall of the putative S1 site and consisted of residues Pro164, Leu167, Leu170, Tyr171, Val172 and Ile173 from the β6/β7 loop, is also conserved in GBSSrtA, where the contributing residues are Pro164, His166, Val167, Val169 and Ile170 (Figure 8a). Therefore, the specificity determining the β6/β7 loop on one side was spatially conserved in the two GBS sortases, and the differences in the β3/β4 loop composition and conformation resulted in a wider active site for GBSSrtA (Figure 8b). In GBSSrtA, where a lid is absent, the N-terminus and part of the β6/β7 loop of the neighboring molecules covered the putative pocket to some extent.

## Discussion

Since the characterization of the first sortase enzyme in 1999 and the discovery of pili in Gram-positive bacteria in recent years, the sortase-mediated biogenesis of pili has been the subject of much interest and investigation. According to the current model of sortase-mediated pilus assembly, the pilus polymerization catalyzed by a pilus-specific sortase is followed by cell wall anchoring of pilus polymers by the housekeeping sortase [56]. The latter step involves the insertion of a minor pilin at the pilus base, which is then anchored to the cell wall and for both these steps the housekeeping sortase is essential. Attempts to understand the structural correlates that dictate the substrate specificities of sortases responsible for pili biogenesis have resulted in the crystal structures of a housekeeping sortase, SPYSrtA, from *S. pyogenes* [42], three pilus-specific sortases from *S. pneumoniae*, SPNSrtC1–C3 [43], [47], and more recently, an atypical, class B pilus-specific *S. pyogenes* sortase C1 [57] and the *Actinomyces oris* sortase C1 [51].

To understand the molecular basis of pilin sorting and pilus anchoring within a bacterial strain, we investigated the housekeeping sortase, GBSSrtA, and a pilus-specific sortase, GBSSrtC1, from the PI-1 pathogenicity island of *S. agalactiae* SAG 2603 V/R. Either GBSSrtC1 or GBSSrtC2 of this GBS strain is capable of polymerizing GBS80, the pilus shaft of the GBS PI-1 pili [18]. However, the incorporation of minor pilins, GBS52 and GBS104, specifically requires GBSSrtC1 and GBSSrtC2, respectively. GBS104 is the tip pilin [2], but the location of GBS52 is still not clear, as it has not been detected by imaging techniques. Interestingly, an ortholog of GBS52 in strain NEM316, GBS1474, has been observed primarily at the base of the pilus and also randomly along the pilus shaft [8], [18]. It has been suggested that the housekeeping sortase, SrtA, catalyzes the cell wall anchoring of the PI-1 polymers via the pilus base, GBS52, in the same manner that SrtA catalyzes the cell wall anchoring of the PI-2 polymers via GBS150 [34]. We have previously characterized the GBS80 and GBS52 crystal structures [58], [59], the substrates for the GBSSrtC1 and GBSSrtA enzymes and identified a pilin-like motif (IYPKI) present at the junction of the N1 and N2 domains of GBS52, similar to the pilin motif (YPKN) of the major pilin GBS80. This observation possibly suggests a conserved secondary substrate binding site in the active site of GBSSrtC1 that can accommodate both pilins.

If the termination and anchoring of PI-1 pili require GBSSrtA to present GBS52 and resolve the acylated complex of GBSSrtC1 and the GBS80 polymer, we suggest an interesting corollary that GBSSrtA should exhibit cross-specificity with GBSSrtC1 in the primary substrate-binding site for recognizing the GBS52 sorting motif but differ in the second substrate-binding site, as its acyl-enzyme intermediate is resolved by a yet unknown cell wall peptidoglycan precursor. What then determines how the two sortase enzymes selectively recognize their cognate substrates for pilus assembly? In this report, we analyzed the crystal structures of two enzymes that share some common features and substrates and differ in several regions, which may contribute to their substrate specificity and function.

A characteristic feature of the class C sortases is the presence of a lid anchored by the DP(W/F/Y) motif in the putative active site, close to the catalytic residues [43], [47]. The enzyme destabilization and decreased efficiency upon DP(W/F/Y) motif mutation in SPNSrtC1 [48] revealed the need for the active site to be occupied and covered. Confirming this hypothesis, a GBSSrtC1 recombinant in which we truncated the entire lid (helix H1 to the β1 strand) remained unfolded (data not shown). The availability of substrate and inhibitor complexes of sortases has been limited to the SASrtA and SASrtB structures [39], [40], [60], which have helped us to demarcate the enzyme active sites. Because the lid needs to be displaced for active site accessibility and the substrate may have a higher affinity than the lid, we generated the GBSSrtC1-lidM recombinant in which we mutated Cys184 to Ala and the anchor region (KDPYS) to IPNTG, the sorting signal motif of GBS80.

The disorder of the IPNTG residues in the GBSSrtC1-lidM structure was attributed to the loss of specific hydrogen bonds. Because the DP(W/F/Y) motif was dislodged by mutation, the entire elongated groove was potentially available for binding; however, the active site is empty around the catalytic residues. This result underscores the possibility of additional interacting components/partners or structural elements beyond the substrate-sorting motif in dictating both specificity. The enzyme integrity was not compromised in GBSSrtC1-lidM, because the hydrophobic pocket in the active site remained protected (discussed below).

Analysis of the residue distribution covered by the lid revealed that the floor region and the walls of the putative active site of the GBSSrtC1 were studded with hydrophobic residues. The pilus-specific GBSSrtC1 and SPNSrtC1 enzymes showed remarkable conservation of all but two of the residues in this hydrophobic pocket (Figures 4a). In contrast, greater differences were observed between SPNSrtC1 and SPNSrtC2/3. The SPNSrtC2 enzyme, which shares functional redundancy with the SPNSrtC1 enzyme, displayed comparatively fewer differences with SPNSrtC1 than SPNSrtC3. A similar hydrophobic pocket and HB-lid residue present in AORSrtC1 suggests that these structural elements are conserved features of class C sortases (Table 1 and Figure 4a). An exception to this is the SPYSrtC1 structure, which is an atypical class B sortase that functions as a class C enzyme [57].

In the GBSSrtC1 and GBSSrtC1-lidM structures, Leu47 was lodged between the β6/β7 loop (Leu167, Leu170 and Tyr171) and the β3/β4 loop (Leu103) and inserted directly above the Val118 and Leu182 present on the β4 and β7 strands, respectively. Analogous to Leu47, Val55 and Ile84/Ile72 were the HB-lid residues in SPNSrtC1 and SPNSrtC2/C3, respectively. The HB-lid residues were one or two residues apart from the anchor motif and adjacent to another hydrophobic residue on either side that pointed away from the active site. Manzano *et al.*, [43] have referred to similar hydrophobic interactions as being essential for covering the TLXT region of the putative active site, a motif conserved in all of the known sortases. Because the lid was absent in the housekeeping sortases, we suggest that the mode of active site accessibility is different, despite the overall hydrophobic nature of the sortase active sites.

Remarkably identical in both the molecules of the dimer, the SPNSrtC1 lid was snugly threaded in the active site by specific polar interactions. SPNSrtC2 and AORSrtC1 lids have similar conformations, with an ordered hinge region located at the N-terminus of the anchor motif. The GBSSrtC1 lid was characteristically longer and highly flexible around the hinge regions on either end. Despite such differences, lid displacement in general is a two-fold problem, which requires lid anchor displacement and the concomitant protection of the hydrophobic pocket. The energetics involved for lid displacement might be offset by the gains in the enzyme reaction. The differences between the active site and lid residues may explain the substrate specificities between the enzymes, as reflected by the abrogation of RrgB polymerization that has been observed upon swapping the SPNSrtC1 and SrtC2 lid regions [48]. The differences in the hinge regions of pilus specific sortases between pathogens may have a bearing on the mechanism of lid displacement employed by different species. However, investigations are needed to delineate the regions in the substrate beyond the sorting motif that may have bearing on recognition by the enzyme.

Both GBSSrtA with SPYSrtA (PDB 3FN7), having an rmsd of 0.89 Å for 98 Cα atoms, suggests noticeable structural differences that extend beyond the barrel core. Interestingly, both GBSSrtA and SPYSrtA exhibited a well-defined and spatially conserved β6/β7 loop, with minor positional differences due to the Zn coordination in GBSSrtA. In addition to the β3/β4 loop, the active site hydrophobic residues in both structures was also spatially conserved (Table 2), and each hosted a Met residue protruding into the active site hydrophobic patch.

**Table 2:** The hydrophobic residues in the active sites of GBSSrtC1 and the housekeeping sortases.

GBS SrtA	SA SrtA(1T2P)	SPY SrtA(3FN7)	GBS SrtC1
(Met101)	(Ala104)	(Met125)	Leu103
(Ala114)	Ser116	(Ser138)	Val118
(Thr167)	-	Val191	Leu167
(Ile170)	(Val168)	(Ile194)	Leu170
§(Val182)	Ile182	Val206	Leu182
Ile194	Ile199	Ile218	Leu195
(Val162)	Pro163	Val186	Ile162
(Asp171)	(Leu169)	Asp195	Tyr171

Residues in parentheses indicate that the residue does not occupy the same spatial position as the structurally corresponding residue in GBSSrtC1.

§ Sidechain conformations are conserved but not the Cα position.

The catalytic residue arrangement in sortases is novel and unique. Site-directed mutagenesis studies in SASrtA have confirmed the role of Arg and His as catalytic partners for Cys184, and the significance of these residues in sortase-catalyzed pili biogenesis of *S. pneumoniae* has also been reported. However, due to the perceived difficulties of a partnership of Cys with His at a physiological pH (pKa = 9.4 and 7.0, respectively in SASrtA) and the catalytically unfeasible distance between His and Cys (d = 4.84 Å, averaged over three chains for SASrtA) obtained from the crystal structures, there is no single catalytic mechanism for SASrtA that satisfies all of the kinetic and structural findings. The proposed need for Ca^2+^ to stimulate SASrtA enzyme activity is different from the effect of Ca^2+^ on the enzyme activity of SPYSrtA [42], [61], suggesting no universal requirement.

We need to understand the differences between the enzymatic mechanisms of housekeeping and pilus-specific sortases for selective inhibition of specific functions in pili biogenesis. The demarcation of the primary and secondary substrate binding sites is not available, even for SASrtA, in which the conformations of some of the loops (if not all) implicated in calcium and substrate binding are different between the X-ray and NMR structures. These differences have been attributed to crystal and molecular packing constraints in the X-ray structures and to unusual covalently bound substrate mimics used in the NMR investigations. However, crystal structures of the enzyme-substrate complex are essential for structure-based design efforts and to demarcate the structural correlates responsible for both substrate specificity and enzyme efficiency.

## Materials and Methods

### Cloning, Expression, Purification and Crystallization

#### GBSSrtA and GBSSrtC1

The expression, purification and crystallization of GBSSrtA and GBSSrtC1 from strain SAG 2603 V/R have been described previously [49].

#### Mutant of GBSSrtC1 (GBSSrtC1-lidM)

To generate the SrtC1 mutation, C225A, the primers VR-SrtB-C225A-5 (5′- cacgtcaccctattaactgccacaccttatatgataaat-3′) and VR-SrtB-C225A-3 (5′- atttatcatataaggtgtggcagttaatagggtgacgtg-3′) were used for PCR-based site-directed mutagenesis, as described previously [56] with pSrtC1S43-Q260 as the template. KDPYS-IPNTG mutations were generated in a similar manner, using primers VR-SrtB-KDPYS-5 (5′-ggcgaatatccagcgcttatccctaatactggtgctgaacaaaagcaggca-3′) and VR-SrtB-KDPYS-3 (5′-tgcctgcttttgttcagcaccagtattagggataagcgctggatattcgcc-3′).

The GBSSrtC1 lid-mutant plasmid was transformed into M15 [pREP4] cells for expression. Luria-Bertani (LB) broth supplemented with ampicillin (100 µg ml^−1^) and kanamycin (25 µg ml^−1^) was used to grow bacteria with shaking at 37°C, until the OD_600_ reached 0.7–0.8. Protein expression was induced by adding 1 mM isopropyl β-D-1-thiogalactopyranoside (IPTG), and the cells were grown for 20–22 hours. The cells were harvested by centrifugation at 2568× g for 15 minutes (min) at 4°C. The pellet was re-suspended in 20 mM sodium phosphate (pH 7.4) / 500 mM NaCl. The cell lysate, obtained by sonication, was then centrifuged at 48384× g for 30–40 min at 277 K. The protocol used for purification of the lid-mutant was similar to that described for the native protein [49]. Purified protein was dialyzed into 50 mM Tris/100–150 mM NaCl (pH 7.4) and concentrated to approximately 5.2 mg/ml (ε = 14,900 M^−1^ cm^−1^, as determined from the www.expasy.org website). The GBSSrtC1-lidM was crystallized using the hanging drop vapor diffusion method at 4°C with 1 µl of the protein mixed with 1 µl of the reservoir solution and equilibrated against 1 ml of 10% (*w/v*) PEG monomethyl ether 2000 and 100 mM MES (pH 6.4).

#### Se-Met GBSSrtA

The seleno-methionine incorporated GBSSrtA was expressed using the M9 SeMET High-Yield growth media kit (Medicilon) following the manufacturer's instructions. The cells were harvested by centrifugation at 2568× g for 20 min, re-suspended in lysis buffer (50 mM Tris pH 7.4, 300 mM NaCl, 0.1 mM phenylmethylsulfonyl fluoride, and 5 mM β-mercaptoethanol) and lysed using a sonicator. After removing the debris by centrifugation at 48384× g for 30 min at 4°C, the supernatant was loaded onto a Ni-NTA column pre-equilibrated with the lysis buffer. The column was washed with buffer A (50 mM Tris pH 7.5, 100 mM NaCl, and 5 mM β-mercaptoethanol). The protein was eluted with 50 mM Tris pH 7.5, 100 mM NaCl and 300 mM imidazole. The His tag was cleaved, and the protein (in 20 mM HEPES pH 7.2, 100 mM NaCl and 5 mM β-mercaptoethanol) was concentrated to 38 mg/ml and crystallized using previously determined conditions [49].

### Data collection, structure determination and model refinement

#### GBSSrtC1 (Type II and III) and GBSSrtC1-lidM

Diffraction data on the GBSSrtC1 type II and III crystals (space group P2_1_2_1_2_1_ and P3_1_2, respectively) were collected at the SER-CAT 22ID beamline at Advanced Photon Source (APS), Argonne National Laboratories (ANL), Chicago and processed with HKL 2000 software [62]. Details describing the diffraction data collection, processing and scaling statistics for type II and type III crystals, respectively were reported earlier [49]. Diffraction data on the GBSSrtC1-lidM crystals in space group P1 were collected in-house using a RIGAKU (100 mA and 50 kV) generator and R-AXIS IV image plates and processed with HKL 2000. Scaled amplitudes were corrected for anisotropic diffraction distribution using SFCHECK in CCP4. The crystal structure of SPNSrtC1 (PDB code 2W1J; sequence identity with GBSSrtC1 = 56%) was used as the search model for the structure determination of GBSSrtC1 (type II) by molecular replacement methods using MOLREP [63]. COOT graphics software was used for model building [64], and the refinement was performed using CNS [65] and REFMAC [66]. The crystal structures of GBSSrtC1 type III and the lid-mutant were determined by molecular replacement methods using GBSSrtC1 type II structure as the starting model, and the model building and refinement were completed as described above. The refinement statistics for all three models are presented in Table 3.

**Table 3:** Refinement Statistics for the GBSSrtC1 and GBSSrtA structures.

	Type II SrtC1	Type III SrtC1	GBSSrtC1-lidM	GBSSrtA
PDB code	3RBK	3RBI	3RBJ	3RCC
Resolution range (Å)	63.6-2.9	65.2-3.0	25.0-2.5	136.9-3.1
Reflections used in refinement	14102	11452	16668	65303
Number of non-H protein atoms	2954	3054	3023	17810
R_work_ (%)	24.8	24.2	24.7	23.2
R_free_ (%)	28.2	30.1	30.4	29.9
Mean B values (Å^2^)	42.8	52.3	44.8	56.6
Type (and number) of ligands/ions	SO_4_(3)	SO_4_(6)	-	Zn (73)
B factor ligand/ion	60.8	98.11	-	61.2
B factor water	30.7	38.6	35.3	19.1
R.m.s.d in bond lengths (Å)	0.022	0.017	0.018	0.015
R.m.s.d. in bond angles (°)	1.93	1.72	1.73	1.58
Number of residues in allowed region (%)	95.3	93.3	94.8	92.3
Number of residues in disallowed region (%)	0.0	1.0	0.0	0.5

#### GBSSrtA_82–238_

The GBSSrtA crystals belong to the C2 space group with 18 molecules in the asymmetric subunit. GBSSrtA shares a sequence identity of 65% and 34% with SPYSrtA and SASrtA, respectively; however, molecular replacement efforts were unsuccessful. The 157-residue GBSSrtA contains four methionine residues; therefore, the seleno-methionine single-wavelength anomalous dispersion (SAD) method was chosen for phase determination. The crystals of Se-Met GBSSrtA diffracted to 2.9 Å resolution at the NE-CAT 24ID beamline (λ = 0.9792 Å) (Table 4). Diffraction data suffered from significant anisotropy and scaled amplitudes were corrected for anisotropic diffraction distribution using SFCHECK in CCP4. Anomalous maps, generated using SHELX-D [67], revealed a positive electron density for three to four heavy atom sites per molecule. The top fifty selenium sites with peak heights greater than 9 σ were selected for refinement and phasing. The PHASER [68] program was used for MR-SAD phasing, using the poly-Ala beta-barrel core of SPYSrtA (residues 72-203, GBS sortase numbering) for the MR. Phase combination, solvent flattening and density modification using the DM program of the CCP4 suite [69], [70] helped in generating the electron density maps suitable for tracing all of the 18 molecules, whose models were built with COOT [64]. In the final cycles of refinement, both TLS and restrained refinement were used in REFMAC [66]. We set aside 3.0% randomly selected reflections for the test set during the refinement and the structure was refined to an R_work_ of 23% and R_free_ of 29% (Table 3) using NCS restrains.

**Table 4:** Data collection statistics for Se-Met GBSSrtA and the lid-mutant of GBSSrtC1.

GBS Sortase	Sortase A	Sortase C1
Crystal	SeMet Derivative	GBSSrtC1-lidM
No of crystals	1	1
Beamline	NE-CAT 24ID	R-AXIS IV generator
Wavelength (Å)	0.9792	1.541
Detector	ADSC 315 CCD	R-AXIS Image Plate
Crystal to Detector distance (mm)	399.18	150
Rotation range per image (°)	1	1
Total rotation range (°)	0–360	0–360
Exposure time per image (s)	10	300
Resolution range (Å)	50-3.1 (3.2-3.1)	38.55-2.3 (2.38-2.3)
Space group	*C2*	*P1*
Unit cell parameters (Å/°)	238.8, 167.8, 97.9; 90, 94.2, 90	43.5, 49.4, 52.7; 87.9, 70.5, 89.4
Mosaicity (°)	3.86	1.16
Total number of measured reflections	593518 (58553)	68884 (6667)
Unique reflections	136005 (13617)	17654 (1707)
Redundancy	4.4 (4.3)	3.9 (3.9)
Mean I/σ(I)	13.6 (1.9)	12.2 (2.9)
Completeness (%)	100 (100)	96.3 (93.9)
χ^2^	1.59 (1.16)	1.00 (1.32)
R_merge_ (%)	13.6 (78.5)	5.2 (40.2)
Number of molecules in asymmetric subunit	18	2

R_merge_ = Σ_hkl_ Σ_i_ I I_i_ (hkl)−〈I (hkl)〉I/ Σ_hkl_ Σ_i_ I_i_ (hkl), where I_i_(hkl) are the intensities of symmetry-related reflections and 〈I(hkl)〉 is the average intensity over all observations.
